# Competitive HIF Prolyl Hydroxylase Inhibitors Show Protection against Oxidative Stress by a Mechanism Partially Dependent on Glycolysis

**DOI:** 10.1155/2013/598587

**Published:** 2013-12-05

**Authors:** Ann-Louise Bergström, Karina Fog, Thomas Nikolaj Sager, Anne Techau Bruun, Kenneth Thirstrup

**Affiliations:** ^1^Department of Neurodegeneration, Lundbeck A/S, Ottiliavej 9, 2500 Valby, Denmark; ^2^Department of Computational and Analytical Chemistry, Lundbeck A/S, Ottiliavej 9, 2500 Valby, Denmark

## Abstract

The hypoxia inducible factor 1 (HIF-1) is a central transcription factor involved in the cellular and molecular adaptation to hypoxia and low glucose supply. The level of HIF-1 is to a large degree regulated by the HIF prolyl hydroxylase enzymes (HPHs) belonging to the Fe(II) and 2-oxoglutarate-dependent dioxygenase superfamily. In the present study, we compared competitive and noncompetitive HPH-inhibitor compounds in two different cell types (SH-SY5Y and PC12). Although the competitive HPH-inhibitor compounds were found to be pharmacologically more potent than the non-competitive compounds at inhibiting HPH2 and HPH1, this was not translated into the cellular effects of the compounds, where the non-competitive inhibitors were actually more potent than the competitive in stabilizing and translocatingHIF1**α**to the nucleus (quantified with Cellomics ArrayScan technology). This could be explained by the high cellular concentrations of the cofactor 2-oxoglutarate (2-OG) as the competitive inhibitors act by binding to the 2-OG site of the HPH enzymes. Both competitive and non-competitive HPH inhibitors protected the cells against 6-OHDA induced oxidative stress. In addition, the protective effect of a specific HPH inhibitor was partially preserved when the cells were serum starved and exposed to 2-deoxyglucose, an inhibitor of glycolysis, indicating that other processes than restoring energy supply could be important for the HIF-mediated cytoprotection.

## 1. Introduction

HIF-1 belongs to the family of hypoxia-inducible transcription factors (HIFs) involved in the regulation of cellular and molecular adaptation to hypoxia [1]. The three isoforms (HIF-1, HIF-2, and HIF-3) are all heterodimers consisting of a constitutively expressed, stable *β*-subunit and an inducible *α*-subunit. The cellular level of the *α*-subunit is regulated at the protein level where high cellular oxygen concentration results in hydroxylation and subsequent proteasomal degradation, whereas low cellular oxygen concentration results in repression of this degradation [2]. When the *α*-subunit is not degraded, it interacts with the *β*-subunit, and the whole complex is translocated to the nucleus and acts as a transcription factor. 

The hydroxylation and thereby stabilization of the HIF-*α* subunit are regulated by the HIF prolyl and asparagine hydroxylase enzymes, of which the prolyl hydroxylases, HPHs, are the focus of this work. The activity of the HPHs is, in addition to oxygen, dependent on iron and 2-oxoglutarate. The activity of the HPHs can thus be inhibited with small molecules either *indirectly* (noncompetitively) through a reduction in the cellular levels of oxygen, iron or, 2-oxoglutarate or *directly* (competitively) by a compound binding to and blocking the 2-oxoglutarate binding site of the enzyme. Alternatively, small molecules interacting with the peptide binding surface could also inhibit the enzymatic activity, but such molecules have yet not been described. There are three isoforms of the HPHs, of which HPH2 has been suggested to be the central regulator of HIF-1*α* [3]. 

HIF-1 induces transcription of several genes involved in adaptation to lower oxygen and glucose supply by (1) optimization of uptake and utilization of oxygen and glucose, (2) optimization of mitochondrial ATP production, and (3) induction of autophagy/mitophagy [4, 5]. 

In a recent paper, the IC50-values of four different HPH-inhibitor compounds towards the three different HPH subtypes were characterized in a new biochemical assay [6]. The two competitive HPH inhibitor compounds, namely, Compound A (CpdA) and JNJ-42041935 (JNJ) have previously been described [6–8]. Both compounds had IC50-values in the nanomolar range towards all three HPH subtypes [6]. The known iron chelator desferroxamine (DFO) and FG41 which are noncompetitive HPH inhibitor compounds were previously characterized and were less potent as they both showed IC50-values in the micromolar range [6]. The functional potency of the HPH inhibitor compounds are however influenced by the cellular concentration of 2-OG which was found to be high in both SH-SY5Y and PC12 cells used in the present study [6]. We therefore further compared the biological effects of these competitive and non-competitive compounds in two different cell types (PC12 and SH-SY5Y).

## 2. Materials and Methods

### 2.1. Cell Lines

Two different cell lines were used: SH-SY5Y cells (human neuroblastoma) and PC12 (rat pheochromocytoma). Both lines were obtained from ATCC. The cells were grown either in DMEM with 4,5 g/l glucose and 10% FCS (standard conditions) or in DMEM with 0,9 g/l glucose and 2% FCS (starvation conditions). Differentiation of SH-SY5Y cells was performed by using retinoic acid and BDNF for 7 days. 

### 2.2. Chemicals

6-OHDA (H4381), 2-deoxyglucose (D8375), and DFO were from Sigma. FG41, Compound A, and JNJ-42041935 were synthesized at H. Lundbeck.

### 2.3. JC-1 Assay for Mitochondrial Membrane Potential (ΔΨ_mit_)

JC 1 assay (T4069) was purchased from Sigma-Aldrich. This assay is based on a fluorescent probe that is able to switch from emission in the green (530 nm) range when it is freely distributed in the cytosol to emission in the red range (590 nm) when it aggregates inside healthy mitochondria. The uptake and aggregation of the probe are crucially dependent on an intact ΔΨ_mit_, thus the ratio between 530 to 590 nm emission is directly correlated to the ΔΨ_mit_. In brief, cells were pretreated with HPH-inhibitor compound for 3 hours followed by treatment for another 3 hours using 6-OHDA. JC-1 reagent was then added to the cells to an end concentration of 4 *μ*g/mL and the cells were incubated at 37°C for 20 min. The cells were washed twice in warm HBSS and fluorescence was then read at Em 535 nm/Ex 590 nm and Em 485 nm/Ex 530 nm and the 530/590 nm emission ratio was calculated.

### 2.4. ATP Assay

Cell-titer Glo (G7570, Promega) was used to determine the total cellular ATP levels. This assay is based on the ATP-catalyzed monooxygenation of luciferin to luciferase. The cells were then treated as described above and after washing twice with HBSS preequilibrated to 37°C, Cell-titer Glo reagent was added to the wells. The cells were lysed by shaking the plate for 2 min and after 10 min of incubation at room temperature, luminescence was read. 

### 2.5. DA-Release Assay

PC12-cells were treated with the indicated compounds for the indicated times and the level of dopamine in the media was quantified by HPLC as previously described [9].

### 2.6. Immunocytochemical Detection of HIF-1*α*

The cells were treated with HPH inhibitors for the indicated hours and then fixed with 4% paraformaldehyde after a short rinse in PBS. The cells were then washed, permeabilized with 0.05% Tween-20 in PBS, blocked with 1% BSA, and incubated with an anti-HIF-1-*α*-antibody (ThermoFisher) for one hour. After washing in PBS, the cells were incubated with a Cy3-labelled secondary anti-mouse antibody for one hour at RT. Hoecst-staining was used for nuclear identification. Analysis was performed with epifluorescent microscopy.

### 2.7. Cellomics ArrayScan Quantification of HIF-1*α* Levels and Nuclear Translocation

**Figure 1:**
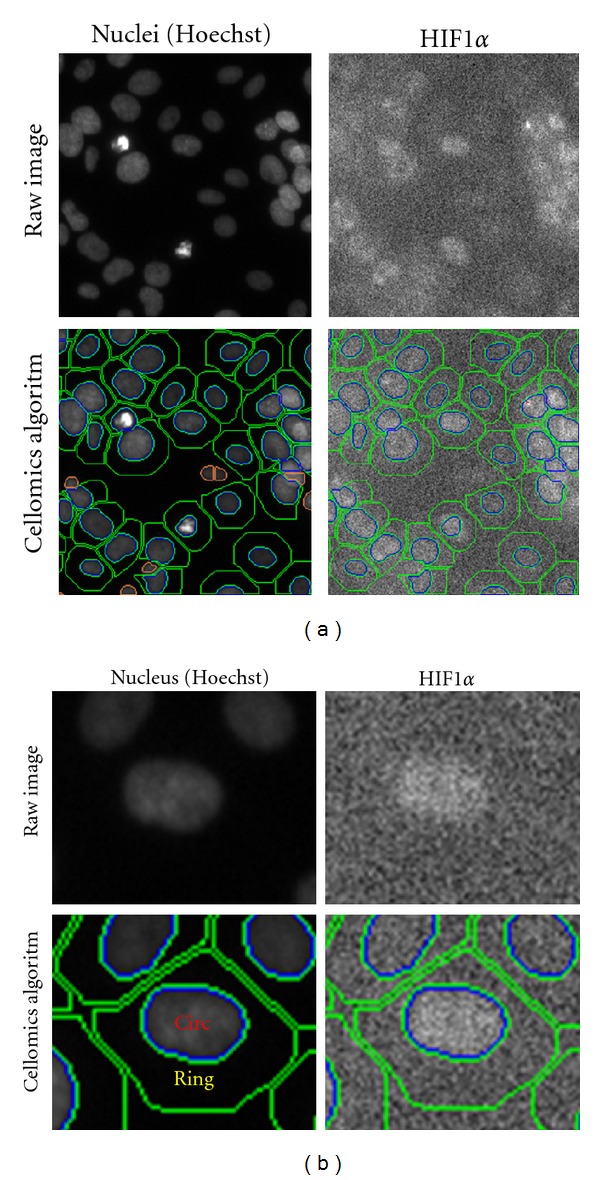
Cellomics images showing the algorithm used for quantification of nuclear and cytoplasmic levels of HIF-1*α*. Raw images without the applied algorithm used to define cells are shown in the top panel and images showing the algorithm are shown in the lower panel. Circ is defined by the outline of the Hoechst-staining and thus represents the nuclear region. Ring is defined as a certain radius surrounding the Circ region and thus represents the cytoplasmic region of the cell. The Hoechst fluorescence and the HIF-1*α* fluorescence are recorded in two different channels.

In order to quantify the level of HIF-1-*α* in the nucleus and cytoplasm, respectively, the Compartmental Analysis BioApplication for Cellomics ArrayScan was used. Cells were grown in 96-well plates, treated with compounds for the indicated times, and fixed with 4% paraformaldehyde. HIF-1-*α* was detected by immunocytochemistry as described above. An algorithm was set up and the level of HIF-1*α*-Cy3-fluorescence in the nuclear region (Circ, see Figure 1(b)) and the cytosolic region (Ring, defined as a fixed diameter region surrounding the nucleus, see Figure 1(b)) was quantified. The ratio of nuclear to cytosolic HIF-1*α* is here representing the level of nuclear translocation of HIF-1*α* when compared to untreated control. 

## 3. Results

Names, structures, and IC50-values for HPH2 binding of the four compounds used in this study are summarized in Table 1. These four compounds were pharmacologically characterized in a recent paper from our laboratory [6].

### 3.1. Both Noncompetitive and Competitive HPH Inhibitors Induce HIF-1*α* Stabilization and Translocation to the Nucleus in a Neuroblastoma Cell Line

First, the capacity of the four compounds to induce HIF-1*α* stabilization and nuclear translocation in the human neuroblastoma cell line SH-SY5Y was analyzed using microscopic analysis and Cellomics ArrayScan quantification. The Cellomics ArrayScan quantification is based on 40 images per well in a 96-well plate with 6 wells per treatment paradigm. Examples of the images are shown in Figure 1(a). Hoechst is used to identify the nuclei (Circ region, Figure 1(b)) and the cytoplasmic region is defined by a user-defined area surrounding the nucleus (Ring, see Figure 1(b)). The levels of HIF-1*α* immunoreactivity levels can be quantified in both of these regions and the ratio of nuclear (Circ) to cytoplasmic (Ring) immunoreactivity can be calculated. Increment of this ratio over time will represent nuclear translocation of HIF-1*α*. 

**Figure 2:**
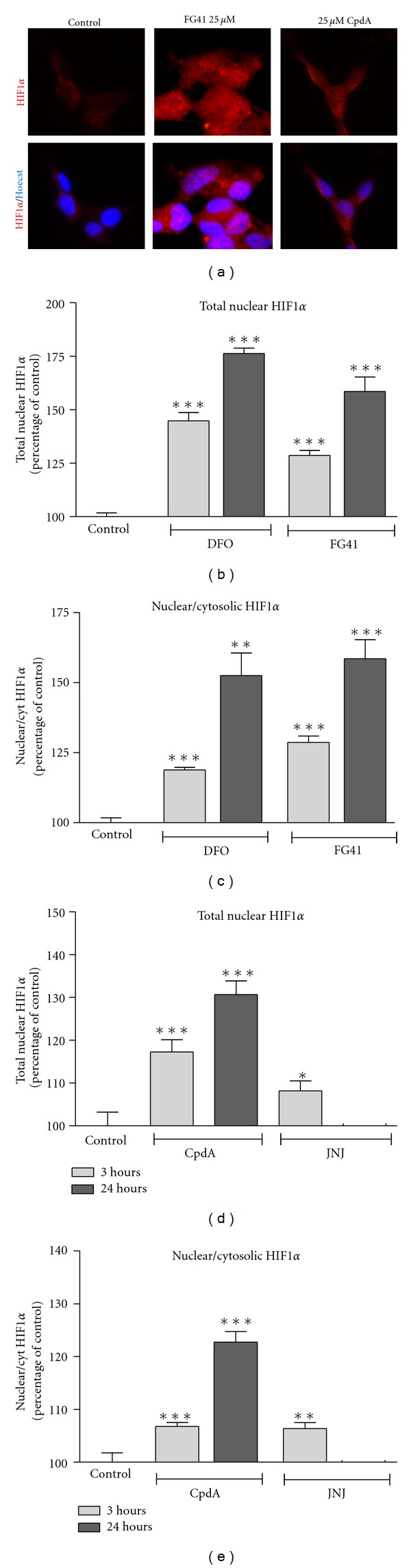
FG41, DFO, CpdA, and JNJ induce HIF-1*α* upregulation and nuclear translocation in SH-SY5Y cells. (a) Immunocytochemical detection of HIF-1*α* in SH-SY5Y cells after 3 hours of treatment with FG41 or CpdA (both at 25 *μ*M). The top panel shows HIF-1*α* immunoreactivity alone and the lower panel shows HIF-1*α* merged with Hoechst. Purple nuclei in the merged image indicate nuclear localization of HIF-1*α*. (b) Cellomics ArrayScan quantification of the total nuclear level of HIF-1*α* induced after 3 or 24 hours of FG41 or DFO treatment (both at 25 *μ*M). The levels are represented as percentage of control (untreated). (c) Cellomics ArrayScan quantification of the ratio of nuclear to cytosolic located HIF-1*α* (percentage of untreated control) after FG41 or DFO treatment as in (b). (d) Nuclear HIF-1*α* after treatment with CpdA or JNJ (both 25 *μ*M). (e) Nuclear/cytosolic ratio of HIF-1*α* after treatment with CpdA or JNJ as in (e). Asterisks indicate levels significantly different from control in a *t*-test.

Using Cellomics quantification, we found that all four compounds were able to significantly increase the nuclear levels of HIF-1*α* as well as the nuclear/cytosolic ratio of HIF-1*α* immunoreactivity (Figures 2(b)–2(e)). This could also be seen in the microscope (Figure 2(a)). There were, however, some differences in the nuclear levels induced by the two categories of compounds. The two non-competitive compounds FG41 and DFO induced higher levels of HIF-1*α* stabilization and nuclear translocation (approximately 75% increase compared to control, Figures 2(b) and 2(c)) than what the two competitive compounds CpdA and JNJ did (approximately 25% increase, Figures 2(d) and 2(e)). Although the timing of the response differed as FG41, DFO, and CpdA all initiated a response that continued to increase from 3 to 24 hours, whereas the response elicited by JNJ was significantly increased at 3 hours but was down to basal level again after 24 hours. 

**Figure 3:**
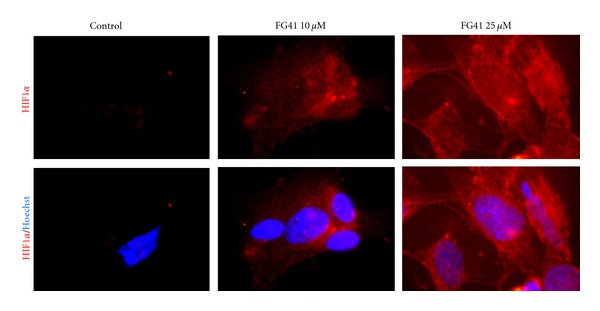
HIF-1*α* stabilization and nuclear translocation in differentiated SHSH-5Y cells. The cells were treated with 10 or 25 *μ*M FG41 for 3 hours, then fixed, and Hoechst-stained. The upper panel shows HIF-1*α* immunoreactivity alone and the lower panel shows the images merged with the Hoechst-staining.

FG41 treatment also resulted in HIF-1*α* stabilization and nuclear translocation in differentiated SH-SY5Y cells showing that the HIF-1*α* stabilization is not only a property of proliferative cells (Figure 3).

### 3.2. Cytoprotective Effect of Competitive and Non-Competitive Compounds

**Figure 4:**
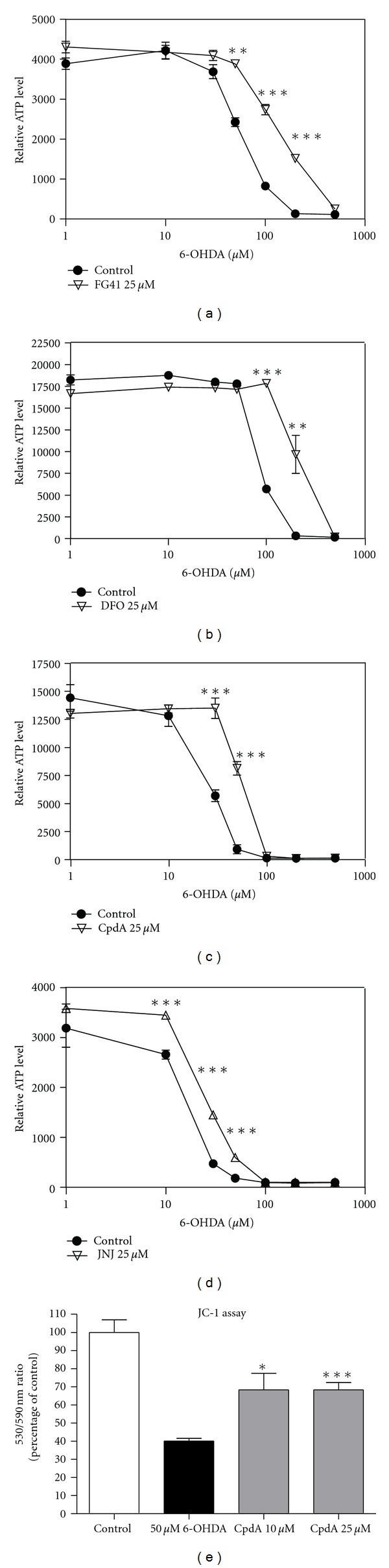
Neuroprotective effect of HPH inhibitors. (a) ATP-levels in SH-SY5Y cells grown overnight in starvation media, pretreated for 3 hours with 25 *μ*M of FG41 (a), DFO (b), CpdA (c), or JNJ (d) followed by 6-OHDA treatment for 3 hours. (e) Mitochondrial membrane potential in SH-SY5Y cells treated with Cpd A (10 or 25 *μ*M) for 3 hours followed by 3 hours treatment with 50 *μ*M 6-OHDA. Asterisks indicate levels significantly increased (*t*-test) as compared to control.

Having established that both the competitive and the non-competitive HPH-inhibitor compounds are able to induce an HIF-1*α* response in the SH-SY5Y cells, we investigated the potential of these compounds to protect against oxidative stress. To model the cellular dysfunction of dopaminergic neurons in Parkinson's disease, where oxidative stress and mitochondrial dysfunction are cellular hallmarks [10], we treated SH-SY5Y cells with 6-OHDA. Treatment with 6-OHDA generates H_2_O_2_ and this oxidative stress leads to collapse of the mitochondrial membrane potential (ΔΨ_mit_) and a reduction in cellular ATP production [11]. We found that 3 hours of 6-OHDA treatment was enough to induce a concentration-dependent collapse of both ΔΨ_mit_ and ATP-production, Figure 4, without reducing the cell numbers (not shown). 

The cells were pretreated with FG41, DFO, Cpd A, or JNJ for 3 hours followed by 3 hours of 6-OHDA treatment in different concentrations ranging from 0 to 500 *μ*M. All four compounds were protective in that pretreatment partially protected against the 6-OHDA induced collapse of ATP-levels (Figures 4(a)–4(d)). This was obtained both when cells were grown in standard media and starvation media (only results from starvation media shown here). We also tested cytoprotection using another assay, the JC-1 assay for mitochondrial membrane potential (ΔΨ_mit_). Also in this readout, there was a trend for the non-competitive inhibitor compounds to be superior to the competitive inhibitor compounds. Using the same timing as in the ATP assay, we obtained significant, but not complete, protection against 6-OHDA induced collapse of ΔΨ_mit_, when the cells were preincubated with CpdA (Figure 4(e)): FG41 or JNJ (not shown). 

### 3.3. HIF-1-Mediated Neuroprotection Is Partially Abolished by Starvation

**Figure 5:**
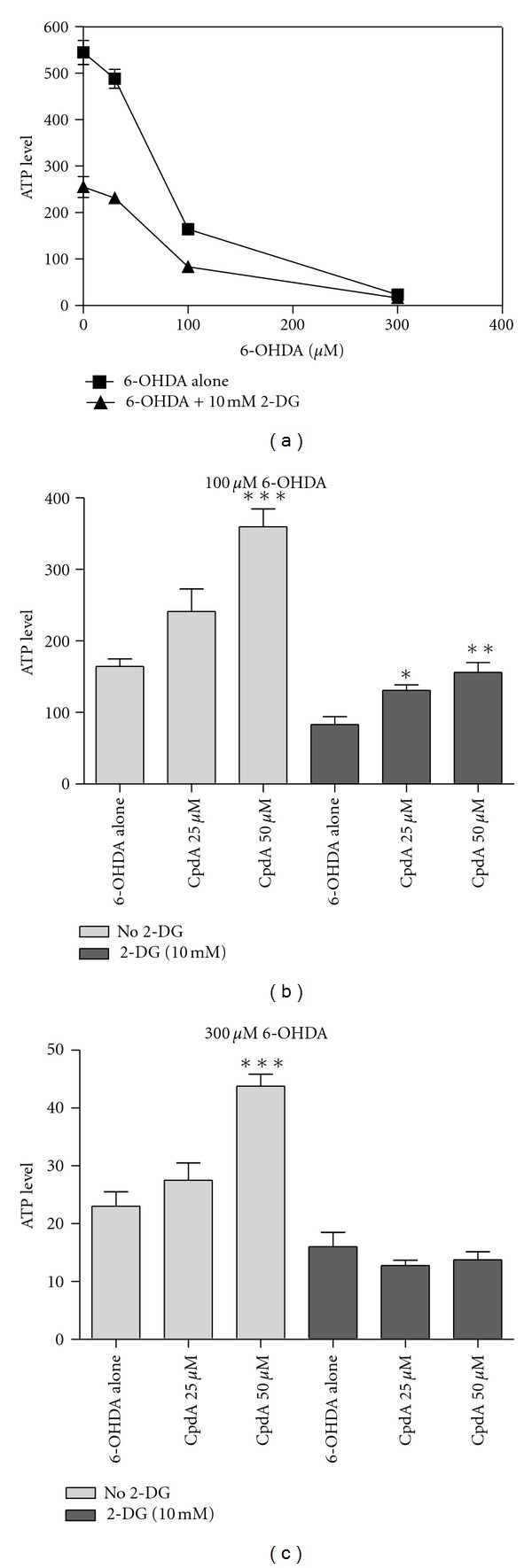
Cpd A is partially protective even when glycolysis is inhibited. (a) ATP-levels in SH-SY5Y cells grown overnight in starvation media overnight, then treated with 10 mM 2-deoxyglucose for 3 hours (or nothing for control) followed by 3 hours of treatment with 6-OHDA at the indicated concentrations. (b) and (c) ATP-levels in SH-SY5Y cells grown over night in starvation media, then pretreated for 3 hours with 10 mM 2-deoxyglucose (or nothing for control) and Cpd A (25 or 50 *μ*M or nothing for control) followed by 3 hours treatment with 100 *μ*M 6-OHDA (b) or 300 *μ*M 6-OHDA (c).

It has been suggested that the primary mechanism of HIF-1-mediated cytoprotection could be ascribed to a metabolic shift towards glycolytic ATP-production [4]. This shift would make the cells less dependent on mitochondrial oxidative phosphorylation and more dependent on anaerobic glycolysis for ATP-production, which would be protective in a hypoxic setting: reviewed by [12]. To investigate this further, SH-SY5Y cells were grown in starvation media (with low glucose and serum levels) for 24 hours and furthermore the cells were treated with 2-deoxyglucose to block glycolysis. Treatment with 2-deoxyglucose significantly reduced both the basal and the 6-OHDA induced levels of ATP in the cells (Figure 5(a)). CpdA was however still able to protect the cells from 100 *μ*M 6-OHDA induced ATP-loss despite the blockages of glycolysis by the combination of starvation media and 10 mM 2-deoxyglucose (Figure 5(b)). The protective effect was relatively similar to what was found when cells were grown without 2-deoxyglucose. At higher doses (300 *μ*M 6-OHDA) the protective effect of CpdA was lost in the presence of 2-deoxyglucose (Figure 5(c)).

### 3.4. HPH Inhibition Induces HIF-1*α* Response and Dopamine Release in PC12-Cells

**Figure 6:**
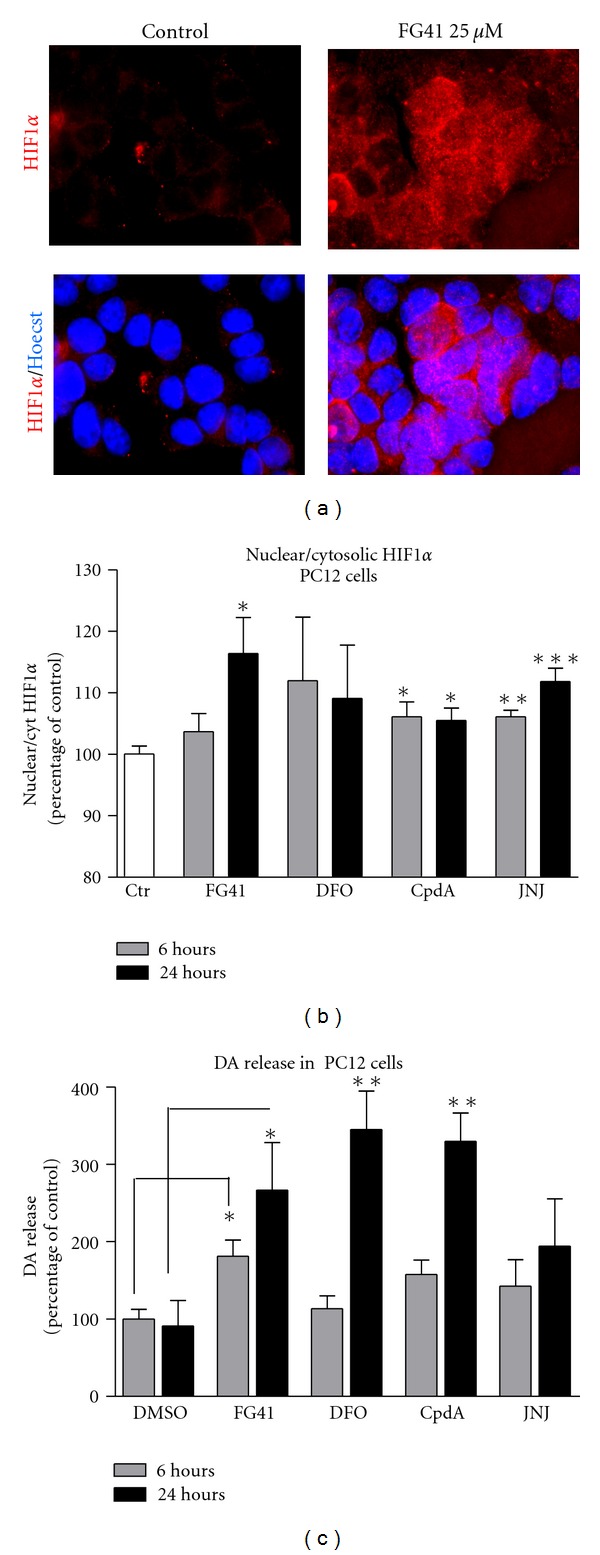
FG41 induces stabilization and nuclear translocation of HIF-1*α* and dopamine release in PC12 cells. (a) Immunocytochemical detection of HIF-1*α* in PC12 cells after 3 hours treatment with FG41 (25 and 50 *μ*M). (b) Cellomics ArrayScan quantification of the nuclear to cytoplasmatic ratio of HIF-1*α* after 6 or 24 hours of treatment with FG41 or Cpd (both used at 25 *μ*M). (d) DA release from PC12-cells induced by 6 or 24 hours of treatment with FG41 or CpdA (25 *μ*M).

We have previously shown that FG41 is neuroprotective in the rat pheochromocytoma cell line PC12 [9]. Using Cellomics ArrayScan and microscopy analysis, we were here able to show that HIF-1*α* could also be induced and translocated to the nucleus in PC12-cells by FG41, which could be seen in the microscope (Figure 6(a)). Using Cellomics quantification, a modest, but however significant, increase in the ratio between nuclear and cytosolic HIF-1*α* levels (thus indicating nuclear translocation) could be induced by both FG41 and CpdA. For FG41, the effect was time dependent in that the ratio was higher at 24 hours than at 6 hours; this was not observed with Cpd A. Despite the rather low HIF1*α* response in these cells, both FG41 and CpdA were able to induce a robust, time-dependent, release of dopamine to the media, Figure 6(c). 

## 4. Discussion

Here we modelled the biological activity of competitive and non-competitive HPH inhibitor compounds by quantifying their ability to (1) induce HIF-1*α*  stabilization and nuclear translocation, (2) protect against oxidative stress, and (3) to induce dopamine release. We found that the biological activity of the competitive HPH-inhibitors CpdA and JNJ was not superior to the non-competitive compounds DFO and FG41 despite the fact that they were previously found to be pharmacologically more active than the non-competitive ones [6, 13]. In fact, the two competitive compounds induced actually a lower and less sustained HIF-1*α* response than what the non-competitive compounds DFO and FG41 did, whereas the downstream effects on protection against oxidative stress and dopamine release were comparable with the two types of inhibitor compounds. Also others have found very efficient stabilization and transcriptional activation of HIF1*α* induced by FG41 and DFO [14–17].

A possible explanation for the observed discrepancy between the pharmacological potency and the cellular HIF-1*α* induction is the influence of cellular 2-OG. The HPH co-factor 2-OG is a TCA cycle intermediate and the levels of this is relatively high in metabolically active cells. The competitive inhibitors used in this study act by binding to the 2-OG site of the HPH enzyme thus competing with the endogenous, intracellular 2-OG. As the cellular concentration of 2-OG in PC12 and SH-SY5Y cells is around 2–3000 *μ*M [6], which is 2000-fold higher than the reported *K*_*m*_ for 2-OG towards HPH2 [18], the HPH inhibition and resulting HIF1*α* upregulation of the competitive inhibitor compounds will be expected to be reduced in these cell types. 

Another factor influencing the biological potential of the two different inhibitor types is iron, which is also a cofactor of HPH. The experiments here were performed in DMEM media in which the iron concentration is comparable to the reported *K*_*m*_-value of iron for HPH2, around 0.3 *μ*M [7]. The combination of an iron concentration that is comparable to the *K*_*m*_-value with the fact that both DFO and FG41 have a high affinity for iron ensures that these compounds effectively are able to inhibit HPH in these experiments [19]. Iron is not only a cofactor of the HPHs but also of the HIF asparagine hydroxylases, meaning the non-competitive inhibitors could be proposed to inhibit both these classes of enzymes, which could be an explanation for the higher and more sustained HIF1*α* response observed with the non-competitive compounds.

Both classes of inhibitors were able to protect the cells against the 6-OHDA induced collapse of ATP-production and mitochondrial membrane potential; this was observed both when the cells were grown in standard media and when they were grown in starvation media with low serum and glucose. There was a trend for a correlation with the induced HIF1*α* level in that also in this parameter, as the non-competitive compounds seemed to be superior to the competitive ones. 

We found that at exposure to a high concentration of 6-OHDA (300 *μ*M), the protective effect of pre-treatment with the competitive HPH-inhibitor CpdA was dependent on a metabolic shift towards glycolytic metabolism, in that glucose and serum starvation combined with 2-DG treatment completely blocked the protective effect. In contrast, when the cells were exposed to a lower dose of 6-OHDA (100 *μ*M), the protective effect was retained. It has repeatedly been shown that HIF-1 stabilization can, in addition to optimizing mitochondrial function [4], induce a metabolic shift resulting in a decreased aerobic, mitochondrial ATP-production via TCA and an increased nonmitochondrial, anaerobic ATP-production via glycolysis, which reduces the oxygen and glucose requirements [20–23]. Thus, at the low dose of 6-OHDA, the optimization of the mitochondrial processes induced by HIF-1 may be sufficient to ensure ATP-production, whereas at the higher dose of 6-OHDA, HIF-1 induced optimization of mitochondrial processes is not sufficient and the cell is thus dependent on glycolytic ATP-production instead. Also other cytoprotective mechanisms could be involved, that is, autophagy, which has also been suggested by others [4, 24–26].

In the PC12-cells, we found that both FG41 and CpdA were able to stabilize and translocate HIF-1*α* to the nucleus, although the levels were lower than what was observed in the SH-SY5Y cells. In the PC12-cells, the non-competitive FG41 induced higher levels of HIF-1*α* induction and nuclear translocation than the competitive CpdA; both compounds however induced a robust and comparable dopamine-release response indicating that either the low level of HIF1*α* is sufficient for the dopamine response or that also other factors than HIF-1 are involved in the regulation of dopamine synthesis and release. 

HIF-1*α* stabilization via HPH inhibition has been shown to upregulate genes relevant for neuroprotection, which induces neuroprotection both *in vivo* and *in vitro* [24, 27–34]. Also in nonneuronal cell types, HPH inhibition has been shown to be protective [4, 35–37]. Prior studies in our laboratory have shown that the HPH-inhibitor FG41 is protective against 6-OHDA induced collapse of mitochondrial membrane potential and cell death in PC12 and LUHMES cells [9]. In addition to the cytoprotective effects of HIF-1*α* upregulation, HIF-1*α* induction also leads to upregulation of tyrosine hydroxylase (TH) and increased level of K^+^ induced dopamine release both *in vivo* and *in vitro *[9, 38]. HIF-1*α* stabilization via HPH inhibition could thus be a potentialstrategy to upregulate therapeutically interesting genes relevant for neuroprotection, vascularization, and dopamine synthesis and it could have therapeutic potential in, for example, Parkinson's disease [29]. Using specific and competitive inhibitors of HPH instead of non-competitive, iron-chelating compounds would be attractive to avoid unwanted side effects as a result of disturbed iron homeostasis; however, our findings here show that the use of such competitive inhibitors could be more complicated than previously anticipated as the cellular 2-OG concentration greatly influences the biological activity of these compounds [7]. If competitive HPH-inhibitor compounds would be aimed for use in Parkinson's disease for the dual purposes of increasing cell survival and dopamine synthesis, it could be a problem that the 2-OG concentration is 4-fold higher in both substantia nigra and striatum compared to cortex. Also other factors such as bioavailability and half life is proposed to influence the biological activity of HPH-inhibitor compounds and make pharmacological activity difficult to translate into *in vivo* therapeutic effects. 

## Figures and Tables

**Table 1:** Overview of HPH inhibitors used: chemical structures and HPH specificity.

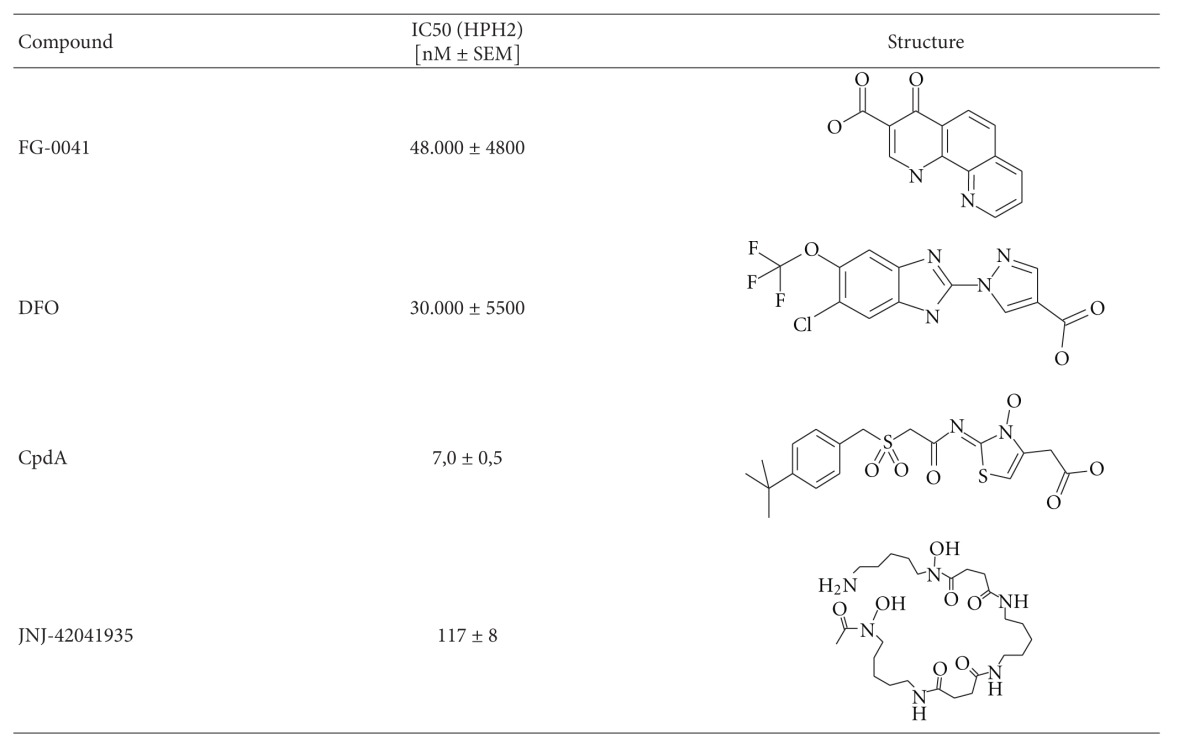

## Abbreviations

HIF-1*α*: Hypoxia inducible factor 1 alpha
HPH: HIF prolyl hydroxylase
6-OHDA: 6-hydroxy dopamine
DFO: Desferroxamine
2-DG: 2-deoxyglucose
2-OG: 2-oxoglutarate.
